# Ethics, Evidence Based Sports Medicine, and the Use of Platelet Rich Plasma in the English Premier League

**DOI:** 10.1007/s10728-017-0345-7

**Published:** 2017-07-29

**Authors:** M. J. McNamee, C. M. Coveney, A. Faulkner, J. Gabe

**Affiliations:** 1 College of Engineering, Swansea University, Swansea, UK; 2 De Montfort University, Leicester, UK; 3 University of Sussex, Brighton, UK; 4 Royal Holloway, University of London, Egham, UK

**Keywords:** Ethics, Evidence based medicine, Placebo, Professionalism, Sports medicine

## Abstract

The use of platelet rich plasma (PRP) as a novel treatment is discussed in the context of a qualitative research study comprising 38 interviews with sports medicine practitioners and other stakeholders working within the English Premier League during the 2013–16 seasons. Analysis of the data produced several overarching themes: conservatism versus experimentalism in medical attitudes; therapy perspectives divergence; conflicting versions of appropriate evidence; subcultures; community beliefs/practices; and negotiation of medical decision-making. The contested evidence base for the efficacy of PRP is presented in the context of a broader professional shift towards evidence based medicine within sports medicine. Many of the participants while accepting this shift are still committed to casuistic practices where clinical judgment is flexible and does not recognize a context-free hierarchy of evidentiary standards to ethically justifiable practice. We also discuss a tendency in the data collected to consider the use of deceptive, placebo-like, practices among the clinician participants that challenge dominant understandings of informed consent in medical ethics. We conclude that the complex relation between evidence and ethics requires greater critical scrutiny for this emerging specialism within the medical community.

## Introduction

Innovative or novel treatments have traditionally been part of sports medicine, where athletes, coaches, and trainers have sought to find a competitive edge over their competitors for performance enhancement, injury prevention, or therapy and return to play. This paper presents part of a more general study whose aims were to: critically explore sports stakeholders’ perspectives on regenerative medicine; describe and evaluate decision-making strategies about musculoskeletal injuries to elite sports performers, and the ethical issues arising in the context of time-related pressures set against considerations of long term athlete welfare. We focus here on the apparent problem of a lack of robust evidence base for these novel treatments, while critically evaluating the rationales offered for their continued use. In doing so we raise complex questions about the relationship between ethical considerations and the clinical relation between healthcare provider—whether physiotherapist, doctor or surgeon—and elite footballers and clubs in the English Premier League (EPL), in the context of attempts to establish a robust ‘evidence base’ for medical practice in sports medicine.

### The Transitional Nature of Evidence Based Medicine

Sports medicine can be understood as a branch of medicine which is maturing as a recognised speciality within the field of medicine [26]. Like many professions it is subject to ongoing issues of legitimation. Some of the less reputable practitioners in its field have been likened to “snake oil salesmen” [19]. It is unsurprising that its leading practitioners and bodies would look to embrace broader legitimising tools such as those to be found in “evidence based medicine’ (EBM). The rise and significance of EBM has been the subject of philosophical and sociological analysis and it is useful to summarise key aspects of this here as it constitutes the backdrop for current debates about different forms of ‘evidence’ in medical science and medical practice. These issues appear, arguably in an extreme form, in sports medicine (understood in a broad sense to include sports physiotherapy).

Evidence-based medicine was originally formulated as an approach combining statistical evidence of the population-level performance of healthcare interventions with qualitative clinical judgment. As its originators noted in their seminal book [55: 71]: ‘It’s about integrating individual clinical expertise and the best external evidence’. In recent debate and medical education it has become primarily associated with statistically-driven research evidence. EBM has seen the development of sophisticated quantitative techniques of meta-analysis to extract the greatest statistical power from scientific studies, usually Randomised Control Trials (RCTs), with the aim of developing robust guidelines on which practitioners can base clinical practice. EBM is thus a medico-political standardization movement for clinical practice [59]. Nevertheless, scientific evidence of the ideal type is usually incomplete and thus clinical algorithms are difficult to derive without other forms of expertise. Hence, especially highlighted has been clinical resistance to EBM, evident for example in what has been called ‘segmental conflict’ between medicine as ‘art’ or ‘science’, and as ‘practice’ or ‘evidence’ [50]. Further, an early analysis of the emergence of EBM suggested that the movement was a defense against encroaching managerialism in health care, and that it could be justified in terms of traditional medical ethics [25]. These conflicts and organizational tensions, as we will show in this article, are reproduced in the sports medicine field. Writing in a *British Journal of Sports Medicine* editorial in 2001, it was opined by one of its leading lights that ‘sports medicine, for so long an art, is slowly but surely turning into a science…How do we progress further? Research, research, research’ [42: 79]. Of course, what matters to the development of a medical specialty is not only the quantity of its research evidence base but also its quality. This is precisely the point at which a careful consideration is needed about which kinds of evidence base is justifiably adopted.

It might be thought that the development of EBM is a linear one: a trend toward ever increasing reliance by clinicians on research driven decisions. While this may be the perception of some sectors of sports medicine, it is not the case for the evidence based medicine movement itself. Contrast the emphasis in the original 1992 definition of EBM:

Evidence based medicine de-emphasizes intuition, unsystematic clinical experience, and pathophysiological rationale as sufficient grounds for clinical decision making and stresses the examination of evidence from clinical research [16: 2420]

with the second version in 1996:

Evidence based medicine is the conscientious, explicit and judicious use of current best evidence in making decisions about the care of individual patients. […] integrating individual clinical expertise with the best available clinical evidence from systematic research [55: 71]

Within this patient-clinician duplex, it is not clear whether the second version de-emphasizes research evidence or re-emphasizes the traditional place of clinical judgement. The net effect is the same. In a third incarnation, and in keeping with the shift toward respect for patient autonomy as a critical ethical norm, we find a triplex of evidence, judgement and patient values constituting EBM:

Evidence, whether strong or weak, is never sufficient to make clinical decisions. Individual values and preference must balance this evidence to achieve optimal decision-making and highlight that the practice of EBM is not a “one size fits all” approach [58: 7].

In summary, there has been a move from a strong emphasis on statistical research evidence to a pluralistic recognition of other sources that comprise EBM. What will be interesting to mark in the following discussion then is the commitment, informed or otherwise, that sports medicine practitioners have to which version of EBM, and how this influences treatment decisions in the elite football world of the English Premier League.

## Methods

The study was multi-method and qualitative in nature.^1^ The football case study comprised 38 semi-structured interviews with a highly specialised group of practitioners and stakeholders mainly in the UK, and a wide range of documents including scientific articles and biotechnology company and product profiles, and observations at specialist international football medicine conferences in the UK, Italy, India and Denmark. Research ethics approval was granted and particular attention was paid to issues of anonymity and confidentiality of interviewees, given the restricted participant pool, in order to avoid identification not only of the source of the data but also the professional players whose treatments and conditions should not be identifiable from the data. The interview sample comprised heads of medical services, deputy heads, Academy heads, or head physiotherapists of English Premier League (EPL) football clubs, representatives from a number of sports and Sport and Exercise Medicine (SEM) governing bodies, companies providing biological therapeutic products, orthopaedic surgeons in the NHS and in private practice both in and outside the UK, medical insurers and advisers. Orthopaedic surgeons and company representatives were identified through their participation at leading sports medicine conferences and approached directly (either in person or via email) by a member of the research team. Doctors and physiotherapists working in the EPL were accessed through an existing network of contacts, facilitated by the project advisory group, and approached via email or telephone. Insurance company representatives and those affiliated to sports governing bodies were identified through web searches and approached via email. They arise from the medical services within the English Premier League football clubs in the UK, surgeons who were contracted to work for them between 2013 and 16 and others from closely affiliated professions. The interviews were analysed according to conventional content analysis techniques, coding the interview data using *Nvivo* software. Our content analysis derived the following overarching themes: conservatism versus experimentalism in medical attitudes; therapy perspectives divergence; conflicting versions of evidence; subcultures; community beliefs/practices; and negotiation of medical decision-making.

## Data Analysis

A range of biological and regenerative techniques were discussed by participants. The most common therapies reported were injections and included platelet-rich plasma (PRP); prolotherapy; ‘Traumeel’; and ‘Actovegin’.^2^ *Traumeel* is claimed to be a ‘natural’ homeopathic treatment^3^ that relieves– pain and inflammation in musculoskeletal conditions. *Actovegin* 4 is made from an ultra-filtered extract of calf’s blood and is used medically (sometimes in conjunction with *Traumeel*) to treat muscle strains. *Prolotherapy* involves sclerosant injections to ligaments, claiming to strengthen/tighten them by provoking an inflammatory response. While different ingredient combinations are used in prolotherapy, most include dextrose or glycerine. *PRP* is the most widely studied of these techniques currently, and the most frequently discussed “novel” treatment used in the EPL. The use of PRP involves extracting blood from the patient, treating it in a high-velocity centrifuge to separate components, resulting in a high concentration of platelets and growth factors and then re-injecting. PRP is thought to aid the healing process by promoting cell growth when re-injected. It is used to treat articular and fibrous (meniscal tear) cartilage injuries, and tendon and ligament injuries. Given that PRP was the most widely discussed treatment, it is the focus of reporting in the present study.

### Conceptual, Empirical and Ethical Problems for Novel Treatments

The different therapies noted above were grouped under what might be called “novel treatments”. Though the term is a vague one, we eschewed the term “innovative” since prolotherapy, for example, has a history of use going back to the late 19th century, though it could not reasonably be described as a standard treatment despite that longevity. The word “novel” therefore came to designate a group of treatments that were used without a robust epidemiological evidentiary basis.

It should be noted that each of the therapies discussed has its own more or less limited evidence base in scientific terms, although in the last decade certainly there have been more studies of PRP than of the others. Moreover, one may reasonably ask what kind of evidence is relevant to professional athletes, and how clinicians evaluate testimony over methodologically robust scientific studies. Nevertheless, a number of problems arise for any novel treatment. These problems might be classified as (1) conceptual; (2) empirical; and (3) clinical. Each will be discussed, before we turn to the ethical issues associated with PRP use.

#### Conceptual Problems

The conceptual problem with PRP is that the various studies and clinical interventions are not comparable. Thus interventions simply designated by the ‘PRP’ concept differ substantively. Many studies are not commensurable since they are based on research that have: spun the blood for varying times at varying speeds; used different needle sizes, had differing sites of injury and/or site of injection, differing time delays after injury; different ages of patient; and so on. This lack of clear definition of the PRP concept complicates any statistical EBM approach through meta-analysis or systematic review of RCTs attempting to establish validity and reliability. This heterogeneity also has the potential to undermine confidence in informed consent procedures with the patient.

#### Empirical Problems: Competing Research Evidence

There are serious problems with the quality of the evidence base for PRP. The scientific community is unclear as to the efficacy of PRP. As noted above, the studies entail conceptual and methodological differences that are a confounding factor to consensus. The picture is further complicated by the wide range of clinical indications that PRP is considered for. We chart the trajectory of scientific evidence on PRP below, focusing on review articles, rather than individual studies.

The published literature reports on the use of PRP injections for a wide range of musculoskeletal sports-related injuries, from tendinopathies (e.g. plantar fasciitis; patella tendinopathy; Achilles tendinopathy; lateral epicondylitis), muscle injuries (e.g. hamstring) and ligament and tendon tears (e.g. Anterior cruciate ligament; Rotator cuff; meniscus; Ulnar collateral ligament) to cartilage repair (e.g. cartilage tears and osteochondral lesions). Additionally, research has been undertaken to assess the efficacy of PRP injections in reducing pain associated with osteoarthritis.

As early as 2006, Anitua et al. [3] reviewed the emerging use of PRP as a method of accelerating healing in musculoskeletal injuries in sports medicine. PRP was referred to as a “relatively new biotechnology” that has been a “breakthrough in the stimulation and acceleration of soft- tissue and bone healing”. They drew attention to significant challenges that needed to be addressed:

First, it is necessary to compare the diverse, platelet-rich products available commercially and to determine how differences in their preparation and use affect their final biological efficacy. Furthermore, procedures need to be standardized and additional, well-designed studies and clinical trials are needed to evaluate the potential therapeutic impact of PRP in medicine and surgery [3: 232].

By late 2007, PRP was described as a “commonly utilised technique” in the delivery of growth factors to injured tissue [9], which although having limited evidence to support its use, is “promising in terms of earlier return to play following muscle and particularly tendon injury”. Faced with increasing clinical use of PRP and the desire for more effective and less invasive therapies in sports medicine, recent years have seen continual calls for more robust scientific evidence, typically in the form of RCTs, to assess long term efficacy, safety and effectiveness in the treatment of specific injuries and guide treatment protocols [45, 51, 56]. The general impression, however, was one of cautious optimism; that further research and rigorous studies will yield the evidence base required to guide treatment protocols but clinical use should be approached with caution pending more research into the basic science of PRP and the development of robust clinical trials to assess safety and efficacy [18, 22, 34, 39, 45, 57].

In December 2010 a consensus position paper was published in the leading Sports Medicine journal, the *British Journal of Sports Medicine.* It aimed to review the current evidence base around PRP in order to provide recommendations for clinicians, athletes and individual sports governing bodies [15: 1079]. The authors, led by the then Head of Sports Medicine for the International Olympic Committee, wrote:

The role of PRP in tissue healing and regeneration may open a new area in regenerative medicine, but there remains a large amount of work required toward understanding the mechanism of action of PRP in the regeneration and repair process of a given tissue. Firm recommendations on the effectiveness of PRP in the clinical setting to support the healing processes of muscle, tendon, ligament and cartilage injuries cannot be given. Results of studies on PRP are difficult to interpret, as the methodological quality of published investigations varies substantially. More attention should be paid to methodological quality when designing, performing and reporting clinical trials

The final recommendation of this consensus group was to “proceed with caution in the use of PRP in athletic sporting injuries” [15: 1079].

The current evidence base for the efficacy of PRP in treating sports injuries remains mixed. While some recent RCTs have found no evidence that PRP promotes better clinical outcomes than placebo [24, 31, 35, 40, 53, 60, 64] other RCTs have found PRP to be beneficial in terms of pain scores and functional or structural outcomes [7, 8, 10, 17, 23, 32, 44, 61], although some of these clinical effects were not long-lasting or could be considered marginal [12, 13].

Injury type is also significant here, with benefits shown in the treatment of patella tendiopathy [37], lateral epicondylitis [54] and OA [49] for example, but not in Achilles tendinopathy [11] or hamstring injuries [52]. Overall, the evidence base for PRP across different injury types can be regarded as *inconsistent and uncertain* [2, 20, 30, 36]. PRP is still seen by some as being an “unproven and experimental” therapy [27] with some suggestion that clinical acceptance of PRP has surpassed scientific evidence for its value and efficacy [27, 48]. Those working in orthopaedic sports medicine and related fields continue to call for more high- level scientific evidence, in the form of randomized controlled clinical trials and statistical analysis to justify the widespread use of PRP in sports medicine [12, 38, 49].

Finally, a more recent large-scale literature review of nearly 100 PRP studies, published in 2016, supports the above picture, concluding that:

…the basic principles, mechanism of action and cellular pathways of PRP and intra-articular MSCs in clinical use needs to be better understood… To address this deficiency, more collaborative randomized controlled studies are needed aimed at standardization of cell or PRP preparation, validated outcome scores and prolonged patient follow-up, with continued study of the mechanism at the basic science level [65: 95]

It seems safe, therefore, to conclude from the literature that little significant progress has been made in PRP efficacy research that might support its users who are committed to evidence-based sport and exercise medicine.

#### Clinical Problems: Evidence, Judgement and Practice

Looking more broadly at relevant clinical evidence, it is noteworthy that the major national body in the UK reviewing and evaluating clinical (and cost-effectiveness) evidence about healthcare interventions is the National Institute for Health and Care Excellence (NICE). NICE has not evaluated PRP in the specific context of sports injuries, but it has examined it in the context of treatment for osteoarthritis of the knee, a condition widely prevalent in (former) football players. Concluding that PRP is safe for this indication, it states that the quality of evidence is too poor to support any verdict on PRP’s effectiveness and thus that in the NHS context: ‘this procedure should only be used with special arrangements for clinical governance, consent and audit or research.’ [46].

Against a background of limited quality of research evidence, therefore, it is important to pose the ethical question of why—with high economic value patients such as EPL footballers (where the median salary is currently calculated at US$ 2.4 million, [21]), clinicians might go out on a limb to use an unlicensed and experimental therapy such as PRP. The answers proffered by participants reveal a complex of clinical, ethical, psychological and social factors.

Of course, as noted above, the concept of ‘evidence’ is contested in the philosophy and sociology of medicine. On the one hand in the 2010 consensus statement we have a cautious approval that does not generally support claims to effectiveness, yet it is apparent that some clinicians will say that their testimony and those of trusted colleagues is sufficient evidence for intervention. The role of such word of mouth and personal contacts points to the role of networks, or ‘sportsnets’ [47] in explaining how therapeutic interventions such as PRP come to be employed despite the lack of an evidence base. Clinicians are members of shared networks which support collective, shared behaviours based on trusted personal recommendations. In such a context ‘normal’ EBM-related standards of evidence may be waived.

One surgeon, who is very widely published and whose clinical credentials were equally widely lauded to the research team, remarked:

I find it great watching the same group of people talking about evidence based, evidence based, evidence based, and then practicing something completely different. I’ve never known a specialty talk about evidence based […] quite so much in an area that has the least evidence basis of any specialty I’ve come across. And yet, you watch the trainees come through SEM [Sport and Exercise Medicine] and all they ever talk about is evidence based and then the next paragraph is about how so and so is about to have an injection of substance X that has only been tested on chimpanzees in Tanzania in a Level 4 study (Participant 8, Orthopaedic Surgeon).

While the view might be thought cynical, the surgeon is undeniably eminent in their field. Nevertheless, the notion of an evidentiary gold standard is problematic on its own terms [59] but more problematic still in relation to the elite sports population under consideration. RCTs might be what scientists hold as an absolute methodological benchmark, but they are not a *sine qua non* for clinicians who have in front of them a single individual who is a highly unusual patient of extraordinary capabilities, a facilitative occupational environment, and extraordinary motivation to return to fitness. Rather the question becomes, how does one use various kinds of evidence—scientific and clinical—to evaluate the desirability of a PRP intervention with this particular athlete, and particular injury, set in this specific occupational context?

Clinicians working in the EPL, like other sports physicians, find themselves making judgements about patient welfare in an arena where their services may be uncomfortably divided between their fiduciary duty to the athlete patient [62] and their employer [1, 14, 41, 63]. Yet the problem of matching evidentiary bases of different kinds with the particular athlete with all their own needs, desires and idiosyncrasies is the persisting problem for clinicians in all branches of medicine. As Participant 23, a leading sports physician explains below, the exercise of good clinical judgment is at the heart of sports medicine practice as opposed to medical science:

I’ve seen four cases with anterior inferior tibiofibular ligament (AITFL) injury where we’ve treated that with PRP […] I’m not aware of any studies that have been published in that area, specifically […] and whether or not this had an effect, I don’t know because I haven’t controlled it. I don’t have a series of other people. But what I do know is if somebody came with an AITFL now, so long as they hadn’t done the back of their ankle and had a thoroughly unstable ankle, I’d stick them in a boot, inject them twice and then have them out of the boot at three weeks and I would expect them to back at six weeks because I’ve seen that happen four times in a row […] Sports medicine has got very little high power of research in it… because of the sample size, and where the questions, particularly in novel treatments, we don’t even understand the treatment. I mean, we don’t know what works in PRP. (…) So then you come down to your own opinion and, obviously, your own opinion is not particularly valid because you haven’t got enough cases to be able to develop an opinion, apart from anecdotal experience (Participant 23, sports physician).

What we are left with is something of an impasse that might reflect medical hierarchies, or at least specialist clinical predilections. The eminent UK surgeon quoted was not the only critical voice regarding interventions not supported by a robust evidence base, yet those with an ongoing relationship with an athlete might argue that their clinical judgement is particularised: sensitive to the needs of the situation that this particular athlete with his history (of performance, injury and recovery) presents.

The foregoing sections provide a context of contestedness for PRP use. On the one hand, as noted above, even recent RCTs are equivocal. Despite this fact there appears widespread use of PRP. We have here a situation that Kimmelman [33] has aptly labelled “translational distance”. And there is little doubt that the participant most engaged with PRP from the perspective of basic science recognises, indeed is almost frustrated by, this gap:

If you have a cell culture of cartilage, fibroblast bone cells and you throw PRP over it, it grows there in a cell culture. But, then, you go to the patient and put the same PRP in their tendon or something like that, it doesn’t seem to work and we don’t know why that is […] I think the best thing you can say about PRP is that it probably is about 100 different growth factors in the small bubbles, in the platelets […] And, sometimes, those factors are very good […] they are meant to be there [but] they are meant to be there at different phases. Whereas, we give everything right away and some of them are very bad for the joint, we know that now. There are now probably 10, randomised controlled studies with different PRPs where half of them have a positive effect, the other has no effect compared to placebo (Participant 10, Sports Surgeon).

Between the laboratory bench and the bedside there is a distance or space that must be bridged by wise judgement incorporating the specific circumstances of the case. But the case should be a reasonably strong one; it is reasonably said that the clinician who proceeds to use PRP against a lack of robust evidence must endure the burden of proof against more established, more conservative interventions that take longer. And clearly in professional football, like any other elite sports injury scenario, time is of the essence. With respect to PRP, however, ought we to consider that the distance is too great? To what extent then is this ethically justified? What grounds might the clinician or healthcare team more generally appeal to in support of their use of PRP?

## Ethical Issues Associated with PRP Use

The most widely used model of medical ethical reasoning, sometimes referred to as “principlism” [5] is comprised of four principles: (1) respect for autonomy; (2) beneficence; (3) non-maleficence; and (4) justice. How does PRP fare in respect of these widely agreed upon principles of medical ethics? Broadly, in line with their contentiousness regarding PRP use, we consider the principles in reverse order.

The issue of justice—understood as fair access to medical services—is not significant here since the EPL players are looked after by medical teams that have considerable finances to spend on player rehabilitation and PRP treatments, though not cheap, are certainly not a major expense. Typically, a player’s medical needs represent only a tiny fraction of the value of Premier League players, though this relationship clearly alters as one considers lower professional leagues.

We shall consider potential harms and benefits together (2 and 3 above) since they are typically the components of a benefit to harm ratio. What ratio does the use of PRP generate? First, the benefits are likely to vary from player to player according to their individual profile (not least their injury history) in terms of certainty and magnitude. The 2010 Consensus Statement [15] offered limited support tempered by the lack of reliable evidence. So why go against a quantitative EBM approach that would yield a low benefit and low harm ratio? Other interventions and placebo seem to offer the same benefit. A number of scenarios might be employed by clinicians if the athlete were nearing their career-end; if other treatments to heal a ligament, tendon or muscle have failed. These contexts appear to offer benefit:harm ratios to justify PRP interventions. One must note that any injection may give rise to infections but for the purposes of argument we assume a safe treatment environment.

The final principle of respecting patient autonomy seems unproblematic at first sight. This requires that treatments are authorised by the player, consistent with *their* conception of their own best interests (as opposed to, say, the clinician’s or their agent’s) and arrived at on the basis of reasonable comprehension of the intervention and all alternatives (including non-intervention). The player, for example, says that they want the treatment and not infrequently their agent is desperate to try some intervention that will return their client to work, and the means exist such that the treatment can be accessed. Respecting player autonomy, however, is not synonymous with doing what they ask. Consent demands comprehension not merely desire. There is, thus, one nagging issue under consent that must be noted. To what extent is the player reasonably informed? The issue here might branch out to issues of autonomy (as voluntary and informed choice) if the player is being pressurised by their agent to seek the swiftest return to play possible. Many of our participants noted that the pressures on attempting novel treatment were often driven by agents who stand outside the therapeutic context, and who were swayed by what they perceived to be effective treatments for other players, despite their lack of medical knowledge of those player-patients or the treatment they had undergone. Such agents may well have a conflict of interest regarding player welfare and their own income generating capacity while a player is ‘on the bench’ or facing long- term injury away from the sport. Finally, with respect to being informed, players’ comprehension of the pros and cons of PRP is problematic in relation to a very mixed epistemic evidence base.

### Casuistry and Control, Paternalism and Placebo

Principlism was developed to counter casuistry. A common appeal to the same ethical principles ought, ceteris paribus, to bring clinical practice into some kind of standardisation that would underpin professionalism. Though contested the principles framework has a hegemonic grip of Anglo-American medical ethics—indeed it was widely referred to as the “Georgetown mantra”^5^ [4]—and has achieved a certain normalisation of status in clinical practice. Despite this hegemony, others have championed casuistry observing that while there may be considerable dispute about ethical theory in medical ethics, there is often widespread agreement in particular cases [29].

Despite its long provenance back to the early Greek and Roman physicians [6] Sports Medicine is a relative newcomer to the professionalisation of western medicine [44]. Moreover, established members of the sports medicine and leading international bodies consistently underwrite their activities as being “evidence-based”. Yet we have noted above the problems for PRP in this regard. Consider, then, the following remarks of one clinician at a leading club. He makes reference to a controversial and indeed notorious (amongst many sports medicine practitioners) treatment undertaken by a practitioner in Belgrade, Mariana Kocavecic, apparently a trained pharmacologist, who provided treatments using a gel incorporating material from horse placenta. Several high- profile players are known to have availed themselves of her services. The clinician indicates the kind of latitude he is prepared to accede in order to secure a speedy return to play:

So, to be honest, if there was somebody next door who was doing horse placenta and the player insisted on it, as long as it wasn’t doing any harm I personally wouldn’t have any problem with it. The simple reason for that is if the player is that fixed in their head that’s always going to be at the back of their mind “oh yeah, but if I had the horse placenta…” So, to try and rationalise it to them and to try and explain to them would be very difficult (Participant 14, Sports physician).

Set against such latitude one could expect few qualms about PRP use. The general evidence-based criticism of PRP may be thought of, in effect, as a criticism of the field more generally. A more pointed criticism came from another surgeon/researcher who remarked:

We did a cohort study which we thought demonstrated that there was possibly some efficacy of doing blood injections for patellar tendinopathy and there have been quite a few papers written on the subject of blood and PRP since. And there’s a lot of papers that don’t show much benefit as I’m sure you’re aware […], but it got to the point where PRP was being injected into everything. I’d heard surgeons from Spain saying they just put it in the knee after surgery. Well, isn’t there quite a lot of blood in the knee? […] So there’s an awful lot of snake oil out there, and I’m quite cynical about it. I resist getting trapped into this sort of arms race that you see particularly in elite sport. I think elite sport almost drives it to some extent (Participant 25, Orthopaedic Surgeon).

The notion that clinicians in the field of sports medicine are engaged in a kind of competition to return athletes to play, coming as it does from a recognised figure, will be troubling to the professional bodies in the field. Yet it is far from clear that it applies in the case of PRP. It is of the utmost importance to note that most of the participants in our study were all well aware of the lack of robust evidence base for PRP. Their use of it was not naive, nor did they think it was some miracle cure. Their rationales varied, however, from the need to control the treatment of their players, to a paternalist concern for athlete welfare, and even the notion that the use of PRP was (akin to) a placebo “treatment”. Here is one very experienced leading football doctor talking of the need for control:

If I have a player and I am offering him a treatment for something which is a problem that he has, in some respects, what it does is it stops him going and getting a treatment from somewhere else because he hasn’t been offered it by me. And I would rather control the degree of mismanagement (Participant 23, Sports Physician).

One can think then, that this is a carefully thought out harm minimisation strategy. In an attempt both to explain and justify PRP against the evidentiary background this physiotherapist remarked about the need to prevent players from seeking alternative treatments and/or places of treatment where the interventions sought might jeopardise player welfare:

What we use these placebo things for is […] to buy ourselves the healing time that we know we need. If I don’t do anything for the player they become frustrated […] It is unacceptable treatment in the eyes of the athlete, in the agents, and everyone else. They will go and seek alternative treatments with that. So, we will then lose. Under the control situation we will do something and […] the mainstay that we tend to do is PRP […] I can’t be having players, behind my back, going to these places, I want to control it. So, we brought it in-house. We sent [our sports physicians] to get the training […] to look at the literature to discuss the various techniques and how we are doing it and what we are doing with it. So, we try to follow as much evidence-based as we can and we’re using the PRP because we consider it safe. We haven’t had any adverse reactions in our knees or any of those issues with it [Participant 11, Sports Physiotherapist].

A joint rationale can be constructed thus: we can control the use of PRP ‘in house’, and it is safe, even if we are uncertain as to its efficacy. This rationale is set against a multitude of players going to (at least) two very well-known surgeons in Germany and Spain. The latter, Dr Ramon Cugat, was referred to in interviews colloquially as the ‘Godfather of PRP’. The former, Dr Hans Wilhelm Muller Wolfhart, gained considerable exposure as the head of sports medicine for Bayern Munich, and also the German national football team (along with many other high profile stars from other sports). But the justification for control is extended into harm *prevention* territory:

I tell you what it’s really done, it’s done a couple of things for us. We will use it in the knee joint, for example. So, the player stops asking for anti-inflammatories because we tell him it’s pointless doing the two because they are going to work against each other. So, suddenly, this dependency and this absolute crush on having to have them, this culture of just pumping, stops. We’ve cut down our anti-inflammatory usage by, it must be, about two thirds, completely gone. So this pre-match “give me an anti-inflammatory, gimme, gimme” has gone (Participant 11, Sports Physiotherapist).

These remarks rendered the most complex justification for the use of PRP. Elements of this rationale were offered by many of the clinical participants in the study in a less sophisticated form. It is multi-layered. It can be analysed under five categories: control of patient care; harm/risk minimisation; player/agent emollient; deceptive; paternalistic. That indeed is a casuistic, though some might argue astute, management of the clinical encounter. Set against player and agent agitation and ignorance, the clinician seeks to achieve the shared outcome—optimal return to play, while managing risks. It is not, in ethical terms however, respectful of autonomy since it represents a level of deception, or at least a lack of full information, for the player-patient. Should this trouble us?

The idea that where a treatment is perceived as being successful it will actively facilitate better patient outcomes—whether objectively or not—is referred to by several other sports medicine professionals, such as this physiotherapist lead of a club’s sports medicine department. But he links it to some ‘shady’ commercial practices by sports medicine companies:

And the players bring them in because they will get tapped up by a company who say “we’ll give you 10 grand [i.e. £10 000], you go and tell all the other guys because we need two or three and once we’ve got that then it will just spiral out, the kids will want them, everyone will want them.” So, fortunately, I’m quite conservative, traditional in that sense rather than I’d like something that’s got more evidence base to it than that. […],if your player really believes in the process of what’s going on, I think the outcomes of that procedure are a lot better and your compliance is a lot better (Participant 11, Sports Physiotherapist).

Now it is clear for many medical interventions that the “buy-in” from the patient to the treatment and their subsequent compliance with rehabilitation protocols is a major factor in successful outcomes. Several participants referred to the use of PRP as if it were a placebo (given their lack of confidence in its efficacy, or their awareness of the patchy evidence base), or where what was enhancing recovery was a “placebo effect” brought about by the patient’s belief system. When pushed on this point, one of the most senior of the medical team participants in terms of experience and status remarked:

That’s where the art of medicine comes in […] we have patients with overload injuries or they are over-trained, maybe, and you’d like to take them out for three months and that’s a lifetime for them. Then, you have to put on something that you do to avert their attention and to get them to do something other than their usual use […] I think this goes on in every aspect of medicine, I think because there are so many things that we think we know but we don’t’ know […] Any clinician will use placebo as part of their medication, so to speak. Any experienced physician (Participant 10, Sports Surgeon).

This is the most explicit defense of PRP as placebo by any of our participants. And this highly regarded clinician expresses a view that the doctor legitimately may deceive the athlete patient in order to arrive at the shared outcome. Nevertheless, questions are raised regarding the extent to which the autonomy of the patient is being bypassed in order to achieve the best outcomes for them. This merits further exploration, beyond the scope of the paper.

## Concluding Remarks

We have attempted to describe the variety of attitudes towards the use of one novel treatment for football injuries in the EPL; the richest football league in the world. It is clear that the world of professional sport, with its exceptional demands on players and the pressures to perform consistently at the highest level means that injuries are a frequent occurrence. One response to these pressures is to seek the shortest route to recovery. On that journey many sets of interests compete: while the player and clinician share goals at one level, such as a speedy, even premature, return to play, they may well have differing views over what is needed to facilitate that (i.e. which treatment modalities) and what the proper time period is. Within sports medicine generally, there has been a significant push towards EBM, without a critical awareness of the shifts in EBM itself. Yet therapeutic “results”, often defined in non-medical terms and by non-medical or health care staff, are being apparently secured in the face of a weak scientific evidence base.

These therapeutic effects are sought with the clinicians’ conception of the players’ best interest in mind. This stands in contrast to the most widely accepted medical principle of respect for the autonomous choices of the patient.

The use of placebo or at least misdirection by some of our sample is a challenging one for any clinician advocating the principles approach to ethical decision-making. Yet our participants appeared untroubled by practices of misdirection and placebo, means that might be thought questionable and certainly at odds with the early statistically dominated conceptions of EBM. While the latter conceptions create greater space or respect for clinical judgment it is not clear that that would extend to deceptive practices. Moreover, it is a moot point whether, and how many, other branches of (occupational) medicine would consider applying the use of the complex strategies that our participants have rationalised here.

These remarks have to be seen in the particular contexts of sports medicine practised in the EPL. Yet there is no good reason to suppose these norms are not to be found in other leagues where the economic considerations are so vast, such as the National Football League or the National Basketball League in the USA. What is difficult to achieve is a reconciliation of these norms and the widespread mantra of EBM. Many of our participants employed, wittingly or otherwise, a casuistic strategy. One may be critical of their reasoning and follow the scornful reminder that one anecdote plus another anecdote does not make evidence [43]. Consequently, the rush to employ PRP in the therapeutic arsenal would be seen as little more than snake oil selling, to use a term favoured by its critics, and a profit-orientated one at that. Or one may follow Jonsen [28], noting how the clinicians worked from received knowledge and iterated between it and their accumulated experience in similar cases, reasoning primarily by analogy to arrive at practically wise particularized judgments. This is not necessarily to exculpate unprincipled clinical decision making, merely to recognize that their commitment to scientific evidence and theory was provisional not absolute. They built up knowledge both of the intervention and of their client and proceeded accordingly.

While one may take opposing views of this strategy it is clear that further reflection on professionalism in sports medicine, the nature and roles of evidence in ethical decision making, and its use in understanding of what constitutes evidence-informed medical expertise is clearly merited.

## Footnotes

1 While two sports formed case studies, football (soccer) and professional cycling in the overarching study, only data from the former are presented here.

2 Although used less commonly, stem cells and autologous whole blood injections were also discussed.

3 It contains ingredients from fourteen mostly flower-bearing plants http://www.traumeel.com/.

4 It is best known for being used by the former Chief Medical Officer for the German football team Dr. Müller–Wohlfahrt.

5 After the University of Georgetown where its authors Beauchamp and Childress taught.
